# How time delay and network design shape response patterns in biochemical negative feedback systems

**DOI:** 10.1186/s12918-016-0325-9

**Published:** 2016-08-24

**Authors:** Anastasiya Börsch, Jörg Schaber

**Affiliations:** 1 Institute for Experimental Internal Medicine, Medical Faculty, Otto-von-Guericke University, Pfälzer Platz 2, Magdeburg, 39106 Germany; 2 Biozentrum, University of Basel and Swiss Institute of Bioinformatics, Klingelbergstrasse 50–70, Basel, 4056 Switzerland

**Keywords:** Stability, Bifurcation, Auto-inhibiton, Mass conservation, p53

## Abstract

**Background:**

Negative feedback in combination with time delay can bring about both sustained oscillations and adaptive behaviour in cellular networks. Here, we study which design features of systems with delayed negative feedback shape characteristic response patterns with special emphasis on the role of time delay. To this end, we analyse generic two-dimensional delay differential equations describing the dynamics of biochemical signal-response networks.

**Results:**

We investigate the influence of several design features on the stability of the model equilibrium, i.e., presence of auto-inhibition and/or mass conservation and the kind and/or strength of the delayed negative feedback. We show that auto-inhibition and mass conservation have a stabilizing effect, whereas increasing abruptness and decreasing feedback threshold have a de-stabilizing effect on the model equilibrium. Moreover, applying our theoretical analysis to the mammalian p53 system we show that an auto-inhibitory feedback can decouple period and amplitude of an oscillatory response, whereas the delayed feedback can not.

**Conclusions:**

Our theoretical framework provides insight into how time delay and design features of biochemical networks act together to elicit specific characteristic response patterns. Such insight is useful for constructing synthetic networks and controlling their behaviour in response to external stimulation.

**Electronic supplementary material:**

The online version of this article (doi:10.1186/s12918-016-0325-9) contains supplementary material, which is available to authorized users.

## Background

Negative feedback is one of fundamental mechanisms in cellular networks [1–7], which fulfils a variety of functions such as mediating adaptation [8–10], stabilizing the abundance of biochemical components [1, 5, 11, 12], inducing oscillations [7, 13–15] and decoupling signal and response time [5]. Negative feedbacks are shown to be present in many biochemical systems including bacterial adaptation [9, 16], mammalian cell cycle [17, 18], stress response in mammalian cells [19] and yeast [20, 21].

Negative feedbacks may involve a time delay, which is needed for signal transduction and transcription, translation and formation of biochemical species [21–24]. The combination of negative feedback and time delay may result in oscillatory dynamics of components of the cellular network [25–27]. Oscillations induced by delayed negative feedbacks (DNFs) were experimentally observed in several biochemical systems as a response to external stimuli and stress, e.g., mammalian Hes1 [24, 28], p53 [29–31] and NF- *κ*B [23, 32, 33] systems.

It is conceivable that oscillatory behaviour might be inappropriate in biological systems mediating adaptive responses. For example, in the hyperthermia treatment of cancer, large-amplitude temperature oscillation could result in tissue damage or patient discomfort [34]. We wondered, if there exist design features and mechanisms of systems containing DNF, which may suppress oscillatory behaviour caused by external stimulation. Recent studies [22, 35] demonstrated that nested negative feedbacks may suppress oscillations of biochemical species involved in DNF. However, these studies provided no insight into how time delay influences the dynamics of DNF systems and interacts with nested negative feedbacks.

In our previous study [36] we derived explicit thresholds and boundaries showing how time delay determines characteristic response patterns of biochemical networks containing DNF. In this manuscript, we continued our research and investigated how the combination of time delay and certain design features influences the dynamics of biochemical DNF systems. To this end, we constructed a range of generic two-dimensional DNF models using delay differential equations. Models differed in several properties:

(i) presence of a nested negative (auto-inhibitory) feedback,
(ii) presence of mass conservation for biochemical compounds,
(iii) mechanism of DNF, i.e., input-inhibition or output-activation.

Further, we subjected these models to computational and analytical stability analyses with respect to time delay. Our computational analyses demonstrate that

- the presence of auto-inhibition and mass conservation have a stabilizing influence on the model equilibrium independent of the DNF strength.
- increasing abruptness and decreasing DNF threshold have a de-stabilizing effect on the model equilibrium.

Our theoretical analyses show that

- nested auto-inhibitory feedbacks may increase the range of time delay, where the system is stable, through the abruptness of the feedback function.

Applying our theoretical framework to the oscillating p53 system in mammalian cells [37] indicates that

- both period and amplitude of p53 oscillations increase with time delay, and
- a nested auto-inhibitory feedback can decouple period and amplitude of oscillations, whereas the delayed feedback can not.

Our analysis provides insight into how time delay and specific design features act in concert to shape the systems dose-response relationship. This knowledge can be used for constructing synthetic networks with the fine-tuned behaviour.

## Methods

### Data

The dataset used for the parametrization of the p53 model was digitized from the supplementary material of [30] from Additional file 1: Figure S6 as described in [22]. It represents an averaged oscillation pattern that was meant to resemble an idealized undamped oscillation with peak characteristics that correspond to the average peak characteristics of oscillating cells.

### Model simulation and analysis

All simulations of the delay differential equations were carried out in Mathematica 9 (Wolfram Research, Champaign, Illinois) using the function *NDSolve* based on the method of steps.

We used DDE-BIFTOOL v. 2.00 [38] and MATLAB R2008b (The MathWorks, Natick, MA) to calculate dependencies between the value of time delay *τ* and amplitude and period of oscillations of the p53 model.

Monte-Carlo analysis was performed in Mathematica 9.

For the parameter estimation we used the least-squares method minimizing the sum of squared residuals (*SSR*):

$$ SSR(p)=\sum_{i=1}^{n} \left(x(t_{i},p)-x_{i}\right)^{2},   $$

where *p*=(*p*_1_,*p*_2_,…,*p*_*m*_) denotes a set of parameters to be estimated, *x*(*t*,*p*) is a numerical solution depending on parameters *p*, *x*_*i*_ is a measured data point at the time *t*_*i*_, *n* is a number of the data points.

For minimizing *SSR*(*p*) with respect to parameter values we utilized the numerical function *NMinimize* in Mathematica 9, which, by default, uses the “Nelder-Mead” method. In case “Nelder-Mead" performs poorly, it automatically switches to the “Differential Evolution” method. The parameter optimization process is assumed to have converged to a local minimum if the difference between the new best and the old best function value *SSR*(*p*), as well as the distance between the new best and the old best parameter values, are less than a tolerance of 10^−8^.

### Robustness with respect to model parameters

We analysed the robustness of the parameter fit for the p53 model with respect to noise. Parameter values were randomly sampled within ±10 % of their respective fitted values using a uniform distribution for 100 times. Then the p53 model was simulated using perturbed parameter sets. The relative variation of the integral of the first transient response after the initial stimulus was calculated (Additional file 1: Figure S6). Namely, we calculated the integral of the simulated p53 model from the time point 0 until the time of the first minimum after stimulation. This way, the robustness of both initial activation amplitude and timing of the first transient response, two characteristic measures of system dynamics, have been estimated concomitantly.

## Results and discussion

### Model formulation

**Fig. 1:**
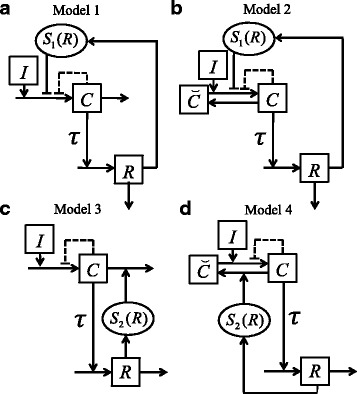
Generic signal-response models with DNF. Squares indicate model variables, circles indicate model functions. Arrows between and to components indicate biochemical reactions, arrows on arrows indicate modifying influences and arrows to functions indicate the respective influence on the function. The models differ in design of the delayed negative feedback (DNF) as well as in presence of mass conservation for the component *C* and auto-inhibitory feedback. **a** Model with input-inhibition as DNF and without mass conservation. **b** Model with input-inhibition as DNF and with mass conservation. **c** Model with output-activation as DNF and without mass conservation. **d** Model with output-activation as DNF and with mass conservation. In all models the time delay *τ* is before activation of the response variable *R*. Dashed lines indicate an alternative auto-inhibitory feedback

We investigated four different two-dimensional models containing DNF that describe generic signal-response networks (Fig. 1). Models differ in design of the DNF and presence of mass conservation for a biochemical compound. All models can have a nested negative (auto-inhibitory) feedback.

We mathematically formulated models from Fig. 1 using deterministic delay differential equations: *Model 1:* DNF with input-inhibition and without mass conservation (Fig. 1a):

1 $$ \begin{aligned} \frac{dC}{dt}=&I\,S_{1}(R)\,F(C)-\alpha\,C,\\ \frac{dR}{dt}=&\,C(t-\tau)-\beta\,R. \end{aligned}  $$

*Model 2:* DNF with input-inhibition and with mass conservation (Fig. 1b):

2 $$ \begin{aligned} \frac{dC}{dt}=&I\,S_{1}(R)\,F(C)\,(1-C)-\alpha\,C,\\ \frac{dR}{dt}=&\,C(t-\tau)-\beta\,R. \end{aligned}  $$

*Model 3:* DNF with output-activation and without mass conservation (Fig. 1c):

3 $$ \begin{aligned} \frac{dC}{dt}=&I\,F(C)-\alpha\,C-\delta\,C\,S_{2}(R),\\ \frac{dR}{dt}=&\,C(t-\tau)-\beta\,R. \end{aligned}  $$

*Model 4:* DNF with output-activation and with mass conservation (Fig. 1d):

4 $$ \begin{aligned} \frac{dC}{dt}=&I\,F(C)\,(1-C)-\alpha\,C-\delta\,C\,S_{2}(R),\\ \frac{dR}{dt}=&\,C(t-\tau)-\beta\,R. \end{aligned}  $$

The function $S_{1}\colon [0,\infty)\to \mathbb {R}_{+}$ is twice continuously differentiable and monotonically decreasing with *R*. The function $S_{2}\colon [0,\infty)\to \mathbb {R}_{+}$ is twice continuously differentiable and monotonically increasing with *R*. The twice continuously differentiable monotonically decreasing function *F*:[0,*∞*)→(0,1] mimics an optional auto-inhibitory feedback.

Parameters of Models 1-4 have real positive constant values. For convenience, we combined them into the vector *p*:

5 $$ p=\left(I,\alpha,\beta,\delta\right).  $$

Note that all model parameters represent lumped biological processes and therefore have only limited physical or biological meaning. For Models 1, 2 the parameter *δ* equals to 0.

In all models the input *I* mimics some constant stimulus (e.g., radiation, see below) that activates a gene transcription network (Fig. 1a, c) or a signalling cascade (Fig. 1b, d) starting with the component *C*. Further, the component *C* activates the response variable *R* with a certain time delay *τ* caused by processes like transport, transcription, translation, etc. Further, the response *R* negatively feeds back through *S*_1_ or *S*_2_ performing the DNF. Depending on the model design the response *R* mediates DNF using different mechanisms.

We refer to models from Fig. 1a, c as a transcription network, because *C* is not reversibly converted into different states, but rather produced and degraded. In the model from Fig. 1a the response variable *R* has the inhibiting influence on *C* by means of the function *S*_1_(*R*). Therefore, we refer to this DNF mechanism as input-inhibition. In the model from Fig. 1c the DNF mediated by the response *R* acts through activating the degradation of *C* by means of the function *S*_2_(*R*). We refer to this DNF mechanism as output-activation. We used this model to simulate p53 protein dynamics [30, 37].

We considered both input-inhibition and output-activation architectures together with a so-called signalling component (see Fig. 1b, d, respectively). The component *C* activating the response *R* originates from another component $\breve {C}$ to which it is constitutively converted back. The sum of both components $C_{t}=C+\breve {C}$ is a constant and assumed to be unity (*C*_*t*_=1). We refer to this model feature as mass conservation. This modelling technique mimics a fast and reversible post-translational protein modification, e.g., phosphorylation, leaving the total protein content unchanged, as it is often described in signalling cascades.

In the following sections we presented theoretical and computational analyses of Models 1-4 with application to p53 system in mammalian cells.

### Stability analysis of systems with delayed negative feedback

Presented in this section stability analysis can be applied to all Models 1-4. Therefore, we do not make an explicit distinction between models, unless necessary.

As the equilibria of Models 1-4 we considered the vector *E*=(*C*_*s*_,*R*_*s*_)^*T*^. The equilibria *E* always exist (see Additional file 1) and implicitly depend on the parameter vector *p* (5). Model equilibria *E* also depend on the input *I* and, therefore, Models 1-4 are not able to show a perfect adaptation.

We linearised Models 1-4 about their respective equilibria *E*:

6 $$ \begin{pmatrix} \frac{d C}{d t}\\[0.7em] \frac{d R}{d t} \end{pmatrix}= \begin{pmatrix} -x& -y\\[0.7em] 0&-\beta \end{pmatrix} \begin{pmatrix} C\\[0.7em] R \end{pmatrix}+ \begin{pmatrix} 0&0\\[0.7em] 1&0 \end{pmatrix} \begin{pmatrix} C(t-\tau)\\[0.7em] R(t-\tau) \end{pmatrix},  $$

where for Model 1, we have

7 $$ \begin{aligned} x&=I\,S_{1}(R_{s})|F'(C_{s})|+\alpha>0,\\ y&=I\,|S_{1}'(R_{s})|\,F\left(C_{s}\right)>0 \end{aligned}  $$

for Model 2, we have

8 $$ \begin{aligned} x&=I\,S_{1}\left(R_{s}\right)\left[F(C_{s})+\left(1-C_{s}\right)\,|F'(C_{s})|\,\right]+\alpha>0,\\ y&=I\,|S_{1}'\left(R_{s}\right)|\left(1-C_{s}\right)\,F\left(C_{s}\right)>0 \end{aligned}  $$

for Model 3, we have

9 $$ \begin{aligned} x&=I\,\left|F'\left(C_{s}\right)\right|+\alpha+\delta\,S_{2}\left(R_{s}\right)>0,\\ y&=\delta\,C_{s}\,S_{2}'\left(R_{s}\right)>0. \end{aligned}  $$

for Model 4, we have

10 $$ \begin{aligned} x&=I\,\left[F(C_{s})+\left(1-C_{s}\right)\,|F'(C_{s})|\,\right]+\alpha+\delta S_{2}\left(R_{s}\right)>0,\\ y&=\delta\,C_{s}\,S_{2}'\left(R_{s}\right)>0. \end{aligned}  $$

The analysis of the model (6) revealed the following stability properties of the equilibrium *E* with respect to *x*, *y*, *β*, *τ* (for details refer to Additional file 1):

- If *xβ*≥*y* holds, then the equilibrium *E* is absolutely stable, i.e., stable for any *τ*≥0.
- If *xβ*<*y* holds, then there exists a marginal time delay *τ*_*m*_ (the Hopf bifurcation point) such that the equilibrium of the model (6) is stable for any 0<*τ*<*τ*_*m*_ and unstable for any *τ*≥*τ*_*m*_. The marginal time delay *τ*_*m*_ is calculated as a product of functions *f* and *g* that depend on *β* and *x*, *y* from (7)–(10), respectively:
  11 $$ \tau_{m}(x,y,\beta)=f(x,y,\beta)\times g(x,y,\beta),  $$
  where
  12 $$  \begin{aligned} &f(x,y,\beta)\\&= \frac{\sqrt{2}}{\sqrt{-x^{2}-\beta^{2}+\sqrt{(x^{2}+\beta^{2})^{2}+4(y^{2}-x^{2} \beta^{2})}}}>0,\\ &g(x,y,\beta)\\&=\arccos{\frac{-(x+\beta)^{2}+\sqrt{(x^{2}+\beta^{2})^{2}+4(y^{2}-x^{2} \beta^{2})}}{2 y}}>0. \end{aligned}  $$
  The derivation of functions *f* and *g* is represented in Additional file 1.

In the next section, we considered features and mechanisms of systems with DNF that may stabilize the system equilibrium after stimulation.

### Design features stabilizing biochemical delayed negative feedback systems

Recently, two research articles indicated that nested auto-inhibitory feedbacks repress oscillatory dynamics of simple biochemical networks involving a non-linear DNF [21, 35]. We wondered how other design features of systems with DNF influence the stability of the model equilibrium. Thus, in addition to auto-inhibition we investigated the influence of the following designs on the stability of the model equilibrium:

- Mechanism of DNF: input-inhibition or output-activation,
- Strength of DNF: strong or weak,
- Presence of mass conservation for a chemical compound.

We also considered how different combinations of delayed and auto-inhibitory negative feedbacks affect the stability of the equilibrium:

- Weak DNF with and without auto-inhibition,
- Strong DNF with and without auto-inhibition.

For analysing the influence of these design features on the stability of the equilibrium we performed a Monte-Carlo analysis of Models 1-4. First, we defined concrete DNF functions *S*_1_ and *S*_2_ and an auto-inhibitory feedback function *F*.

We defined a reverse Hill function as the input-inhibition DNF function *S*_1_(*R*). As the output-activation function *S*_2_(*R*) we defined a Hill function. Thus, functions *S*_1_ and *S*_2_ have the following form:

13 $$\begin{array}{@{}rcl@{}} S_{1}(R)&=&\frac{{K_{m}}^{n}}{{K_{m}}^{n}+R^{n}}, \end{array} $$

14 $$\begin{array}{@{}rcl@{}} S_{2}(R)&=&\frac{R^{n}}{{K_{m}}^{n}+R^{n}} \end{array} $$

with *K*_*m*_>0 being the half-saturation constant, characterizing the activation threshold beyond which the feedback takes effect, and *n*≥1 being the Hill coefficient, characterizing how abrupt the feedback takes effect after having passed the activation threshold. Thus, parameters *K*_*m*_ and *n* specify the strength of the DNF: the lower the activation threshold *K*_*m*_ and the higher the abruptness *n*, the stronger the DNF is. Note that applying the same parameters make functions *S*_1_ and *S*_2_ symmetric, allowing a fair comparison of the influence of input-inhibition and output-activation on the model stability.

As the auto-inhibitory feedback we employed a reverse Hill function *F*(*C*):

15 $$ F(C)=\frac{1}{1+\left(\kappa\,C\right)^{\nu}},\,\nu\ge 1,\,\kappa\ge 0.  $$

**Fig. 2:**
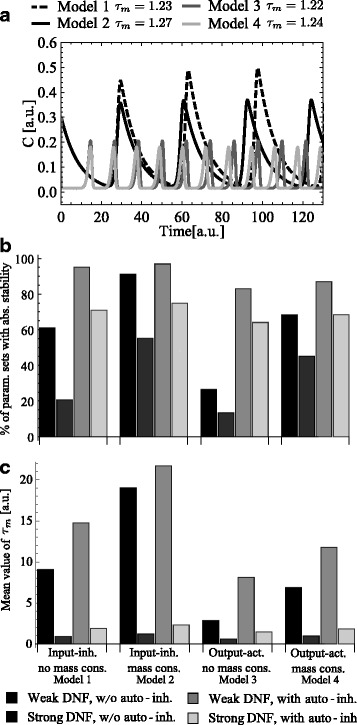
Results of Monte-Carlo analysis of Models 1-4. a Simulation of Models 1-4 with parameter values *I*=0.87, *α*=0.11, *β*=0.17, *δ*=58.2, *n*=12.77, *K*_*m*_=0.23, *τ*=2.5 without auto-inhibition (*κ*=0). **b**,**c** Stability analysis of Monte-Carlo simulations of Models 1-4. Model parameters were randomly sampled 10000 times in the certain range. The range was defined according to assumptions about model characteristics: strength of DNF (strong or weak) and presence of auto-inhibition. The percentage of parameter sets (see Fig. **b**), which induced absolute stability, and the mean value of marginal time delay *τ*_*m*_ (see Fig. **c**) were quantified

Then, we randomly generated parameter values *I*=0.87, *α*=0.11, *β*=0.17, *δ*=58.2, *n*=12.77, *K*_*m*_=0.23 in the way that Models 1-4 without auto-inhibition, i.e., *κ*=0, have similar values of *τ*_*m*_. They correspond to *τ*_*m*_=1.23, *τ*_*m*_=1.27, *τ*_*m*_=1.22, *τ*_*m*_=1.24 for Models 1-4, respectively. Thus, applying this parameter set we guarantee that any value of the time delay *τ* has a similar distance to the Hopf bifurcation point *τ*_*m*_ for all considered models. Simulations of Models 1-4 with these parameters and *τ*=2.5 are depicted in Fig. 2a.

Further, using these parameter values we performed a Monte-Carlo analysis for Models 1-4 with the constant input *I*=0.87. Namely, the rate constants *α*, *β*, *δ* have been sampled in the range from 0.1 to 10 times of their respective values for 10000 times. The parameter values defining the system design, i.e., *n*, *K*_*m*_, *ν* and *κ*, were sampled in the following way:

(i) in the case of weak DNF without auto-inhibition (*κ*=0) we sampled *n* in the range from 0.1 to 1 time of its value and *K*_*m*_ in the range from 10 to 20 times of its value 10000 times.
(ii) in the case of strong DNF without auto-inhibition (*κ*=0) we sampled *n* in the range from 1 to 2 times of its value and *K*_*m*_ in the range from 0.1 to 10 times of its value 10000 times.
(iii) in the case of weak DNF with auto-inhibition we sampled *n* and *K*_*m*_ as in (i), *κ* in the interval [0.1,10], *ν* in the interval [1,20] 10000 times.
(iv) in the case of strong DNF with auto-inhibition we sampled *n* and *K*_*m*_ as in (ii), *κ* in the interval [0.1,10], *ν* in the interval [1,20] 10000 times.

Thus, for each Model 1-4 and each combination of DNF and auto-inhibition we obtained 10000 parameter sets. For each parameter set we calculated *x* and *y* according to (7)–(10) for Models 1-4, respectively. Then, we defined the percentage of parameter sets for which *xβ*≥*y* holds indicating absolute stability of the model equilibrium (see Fig. 2b). For all other parameter sets, we calculated the mean value of the marginal time delay *τ*_*m*_ (11), i.e., the Hopf-bifurcation point (see Fig. 2c).

Figure 2b, c shows that considered models with auto-inhibition have a higher percentage of parameter sets leading to absolute stability and higher mean value of *τ*_*m*_ than the same models without auto-inhibition. This observation confirms previous results [21, 35] showing that nested auto-inhibitory feedbacks repress oscillatory dynamics in networks containing DNF.

Additionally, for models with weak DNF there are more parameter sets, which induce absolute stability of the model equilibrium, than for models with the strong DNF. Accordingly, models with weak DNF have higher mean value of *τ*_*m*_, i.e., are less prone to oscillations, than models with the strong one. Thus, the nested auto-inhibitory feedback and the DNF have opposing effects on the system’s stability. The stronger the auto-inhibitory feedback and the weaker the DNF, the less probable an oscillatory response of the system is.

Moreover, Models 2 and 4 with mass conservation have a higher percentage of parameter sets leading to absolute stability of the model equilibrium and higher mean value of *τ*_*m*_ than respective Models 1 and 3 without mass conservation. In order to explain this effect, for each model we quantified the dependence between *τ*_*m*_ and each parameter value in the range from 0.1 to 10 times of its default value leaving the other parameters fixed (see Additional file 1: Figure S11a–d). This sensitivity analysis shows that the presence of mass conservation influences the sensitivity of *τ*_*m*_ only with respect to the half-saturation rate *K*_*m*_ leaving all other parameter sensitivities unchanged (compare Additional file 1: Figure S11a to b and S11c to d for models without and with mass activation, respectively, and Additional file 1: Figure S11e). In the presence of mass conservation *τ*_*m*_ increases much stronger with increasing feedback activation threshold *K*_*m*_ and, therefore, stabilizes the equilibrium. Moreover, in the presence of mass conservation the value of *K*_*m*_ beyond which *τ*_*m*_ does not exist any more, i.e., the system’s equilibrium becomes absolutely stable, also decreases (Additional file 1: Figure S11e).

Concerning the design of the DNF, Monte-Carlo analysis shows that Models 1, 2 with input-inhibition have a higher percentage of parameter sets leading to absolute stability and a higher mean value of *τ*_*m*_ than Models 3, 4 with output-activation (see Fig. 2b, c). However, we were not able to support these results with an alternative parameter set *I*=0.48, *α*=0.14, *β*=0.44, *δ*=83.71, *n*=10, *K*_*m*_=0.9. Refer to Additional file 1: Figure S12a for simulations of Models 1-4 using the alternative parameter set and *τ*=10. In comparison, for the alternative parameter set Models 1, 2 with input-inhibition have higher values of *τ*_*m*_, i.e., *τ*_*m*_=1.27 and *τ*_*m*_=1.86, than Models 3, 4 with output-activation, i.e., *τ*_*m*_=0.36 and *τ*_*m*_=0.52. Thus, the distance of *τ* to *τ*_*m*_ is smaller for Models 1, 2, and one may expect a higher percentage of parameter sets inducing absolute stability. Nevertheless, for the alternative parameter set the Monte-Carlo analysis showed that models with input-inhibition have approximately the same percentage of parameter sets leading to absolute stability as corresponding models with output-activation. In comparison, all other conclusions presented above were confirmed for model simulations with the alternative parameter set (Additional file 1: Figure S12b).

Taken together, we conclude that auto-inhibition as well as mass conservation have a stabilizing influence on the model equilibrium independent of the strength of DNF and allow systems with DNF to adapt to an external stimulus without producing sustained oscillations. Moreover, the higher the activation threshold and the less abrupt the DNF, the less prone the system is to an oscillatory behaviour.

### Auto-inhibition increases *τ*_*m*_

Computational analysis presented in the above section demonstrated the opposing behaviour of auto-inhibitory and delayed negative feedbacks with respect to stability (Fig. 2). In this section, we analytically investigated how the auto-inhibitory feedback stabilizes the equilibrium of the system.

We proved that *τ*_*m*_ (12) increases with *x* and decreases with *y* (see Additional file 1):

16 $$  \partial\,\tau_{m}(x,y,\beta)/\partial\,x>0,\, \partial\,\tau_{m}(x,y,\beta)/\partial\,y<0 \quad\text{for \(x,\,y>0\).}\quad  $$

Further, we derived upper and lower bounds for *x* and *y* from (7)–(10) for Models 1-4, respectively (see Additional file 1):

17 $$ \begin{aligned} 0<\varepsilon_{lb}(|F'\left(C_{s}\right)|)&<x<\varepsilon_{ub}(|F'\left(C_{s}\right)|),\\ 0\le\sigma_{lb}&<y<\sigma_{ub}. \end{aligned}  $$

Both the lower and upper bound of *x*, i.e., *ε*_*lb*_ and *ε*_*ub*_, are increasing with |*F*^′^(*C*_*s*_)| for Models 1-4. Therefore, we can always increase a given *x* by choosing an appropriate value of |*F*^′^(*C*_*s*_)|. The lower and upper bound of *y*, i.e., *σ*_*lb*_ and *σ*_*ub*_, have non-negative constant values. Consequently, according to (16), we can always increase *τ*_*m*_ by increasing |*F*^′^(*C*_*s*_)|.

Taken together, we showed that auto-inhibitory feedback can increase the range of the interval [0,*τ*_*m*_) and stabilise the model equilibrium.

### Application to the p53 system

In this section we applied Model 3 to the p53 system to gain novel insights into the functioning of this system.

The tumour suppressor protein p53 is activated in response to many stress signals and activates various stress-response programs including cell-cycle arrest, senescence and apoptosis [39]. It is also well established that p53 acts within a negative feedback loop, including Mdm2 as the negative regulator of p53: p53 transcriptionally activates Mdm2, which in turn targets p53 for degradation [29, 40].

Several mathematical models of p53-Mdm2 feedback loop have been published [22, 28, 30, 37]. One of these models (model III from Table 1 in [30]) is a particular case of the model from Fig. 1c corresponding to the Model 3 with *F*(*C*)≡1. Therefore, we wondered, whether our framework would also be able to explain measured p53 dynamics upon DNA damage. In our designations *C* and *R* correspond to p53 and Mdm2, respectively. The input *I* is defined here as a scaled DNA damage signal and is measured in arbitrary units. The negative feedback by output-activation is modelled by a non-linear Hill function *S*_2_(*R*) (14).

**Fig. 3:**
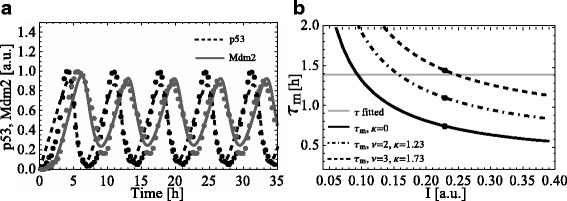
Simulation and response analysis of the p53 model. a Simulation of the p53 model (3) with fitted parameters from Table S1 (see Additional file 1), dots – experimental data from [30], Fig. S6 therein. b Dependence between the stimulus value *I* and *τ*_*m*_ for the p53 model (3) with fitted parameters from Table S1 (see Additional file 1) without and with synthetically activated auto-inhibitory feedback *F*(*C*) (with *ν*=2, *κ*=1.23 and *ν*=3, *κ*=1.73). Dots designate values of *τ*_*m*_ calculated for the fitted value of *I*=0.23 for the p53 model (3) with and without auto-inhibitory feedback

We fitted parameters of the p53 model (3) to the experimental data of an averaged oscillation pattern of the p53-Mdm2 system after DNA damage from [30] (Additional file 1: Fig. S6 therein). The results of the fit are presented in Additional file 1: Table S1. Figure 3a shows the simulation of the p53 model (3) with fitted parameters. The model well recapitulates measured p53 dynamics after DNA damage. Moreover, the fitted optimal solution is also robust with respect to noise in the fitted parameters (see Additional file 1). Indeed, the integrated first transient response varies only by 8.7 % assuming a parameter noise of ±10 *%* (see Methods Section and Additional file 1).

The model analysis shows that the fitted time delay *τ*=1.37 h is almost two times larger than the corresponding *τ*_*m*_=0.76 h that was calculated for the fitted DNA damage signal *I*=0.23 (Fig. 3b). Therefore, the p53 model (3) with fitted parameters from Table S1 (see Additional file 1) produces sustained oscillatory response.

It was earlier reported that distinct p53 dynamics such as oscillations or sustained activation may lead to different cell fate decisions [31, 39]. Recent study [41] showed that the system’s response is modulated by DNA damage strength. Namely, after high DNA damage p53 level was monotonically increased and cells activated apoptosis, whereas after low DNA damage p53 level underwent periodic pulsing resulting in a cell-cycle arrest. We checked if our generic p53 model (3) is able to reproduce this transition with respect to the DNA damage level. Figure 3b shows that *τ*_*m*_ is decreasing with respect to the DNA damage signal *I*. Hence the fitted value of time delay *τ* is greater than *τ*_*m*_ for any *I*>0.23 (fitted value). Therefore, the p53 model (3) produces sustained oscillations for any *I* greater than the fitted value and is not able to perform the transition from oscillatory to adaptive behaviour with respect to increased DNA damage signal *I*. This conclusion is also applicable to other model alternatives (1)–(4) used in our previous study presenting models of HOG pathway in yeast and NF- *κ*B signalling in mammalian cells (see Figs. 3b and 6b in [36]). However, according to [41] the switch from sustained oscillations to monotonic increase of p53 level is regulated by a mechanism attenuating Mdm2 expression that is not present in the current p53 model. Studies [39, 41] considered DNA damage kinases ATM and ATR as negative regulators of Mdm2 expression. Using this knowledge, we extended the p53 model (3) by including the additional component ATM activating p53 and attenuating Mdm2 (see Additional file 1: Figure S7). As a result, the extended p53 model was able to qualitatively reproduce the switch from oscillations to monotonic increase of p53 level (see Additional file 1: Figure S8). Simulations of the extended p53 model suggest that this transition between response types originates from the competition between ATM and p53 for the inhibition and activation of Mdm2, respectively. In case of high DNA damage, ATM level is high and suppresses Mdm2 giving a monotone increase of p53 level. In case of low DNA damage, Mdm2 activity is not effectively suppressed by ATM resulting in sustained oscillations of both p53 and Mdm2.

Further, we applied our theoretical analysis to explore under what conditions sustained oscillations of p53 model (3) can be suppressed by the activation of a nested auto-inhibitory feedback to the model component *C* preserving all values of fitted parameters. Our theoretical analysis suggested that the marginal time delay *τ*_*m*_, beyond which any time delay leads to sustained oscillations, can be increased by increasing the slope of the auto-inhibitory feedback function at the equilibrium |*F*^′^(*C*_*s*_)|. As in the previous section, as the auto-inhibitory feedback function we utilized a reverse Hill-function *F*(*C*) (15). Further, we adjusted parameters *ν* and *κ* of *F*(*C*) and calculated *τ*_*m*_ (see Additional file 1). For *ν*=3 the marginal value of time delay *τ*_*m*_ is larger than the fitted time delay *τ*=1.37 h (Fig. 3b). As a result, p53 model (3) with parameters from Table S1 in Additional file 1 produced damped oscillatory response (Additional file 1: Figure S4).

**Fig. 4:**
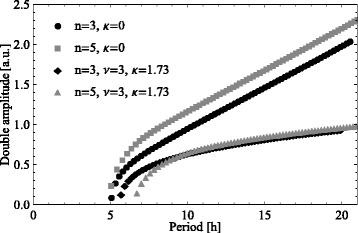
Amplitude/period curves of the p53 model under variation of *τ*. The analysis is performed for the p53 model (3) without and with synthetically activated (*ν*=2, *κ*=1.23; *ν*=3, *κ*=1.73) auto-inhibitory feedback using values of the Hill coefficient *n*=3 and *n*=5 (fitted value) of the DNF function *S*_2_. Period and amplitude were quantified for the time delay *τ* varied in the range from 1 to 8 hours with the step 0.2 hour. Both amplitude and period of oscillations increase with *τ*

In a similar DNF system it was shown that the period of oscillations increases with the Hill coefficient *n* of the DNF function for a given time delay [24]. We wondered how parameters of the delayed negative and auto-inhibitory feedbacks influence the amplitude and period of oscillations in our system. Figure 4 demonstrates that the auto-inhibitory feedback (with parameters *ν*=3, *κ*=1.73) decreases and stabilizes the amplitude of oscillations, whereas the amplitude of oscillations increases with respect to the Hill coefficient *n* of the DNF function *S*_2_. Moreover, increasing the abruptness of the DNF has no substantial influence on the increase of period with respect to time delay *τ*. The period of oscillations is a linear function of the time delay *τ* irrespective of values of *ν*, *κ* and *n*. Thus, opposed to the delayed feedback, the auto-inhibitory feedback has the potential to de-couple the increase of amplitude and period of oscillations with respect to *τ*. Moreover, auto-inhibitory and delayed negative feedbacks have an opposing influence on the amplitude of oscillations.

Thus, our analysis showed that for the p53 model (3) an auto-inhibitory feedback can be a potential mechanism increasing the marginal time delay *τ*_*m*_, decreasing the amplitude of oscillations and turning sustained oscillations into damped oscillations.

The experimental study of p53 oscillations [29] concluded that the mean number of p53 pulses in individual cells increased with DNA damage. Moreover, the authors suggest that the p53-Mdm2 feedback loop generates a “digital” clock making the number of p53 pulses relevant for the cell fate, and not their amplitude and duration. However, this hypothesis has not been proven yet. Therefore, we wondered which parameters of p53 model (3) play a prominent role in controlling the length of p53 pulses. Using p53 model (3) we split the p53 simulation curve on “On” and “Off” states (see Additional file 1: Figure S9). Then we checked how different model parameters control the duration of p53 pulses (see Additional file 1: Figure S10). The analysis showed that time delay *τ* is the only parameter that significantly changed the duration of “On” and “Off” states of the model response (see Additional file 1: Figure S10b). Namely, time delay *τ* increases the duration of “Off” states and decreases the duration of “On" states. In addition, *τ* increases the amplitude and period of pulses (see Fig. 4). Note that the same conclusion can be applied to the relation between time delay *τ* and marginal time delay *τ*_*m*_: the higher *τ*/*τ*_*m*_, the higher the amplitude and period of pulses are. It would be interesting to validate these predictions experimentally and check the physiological effect of changing time delay between p53 and Mdm2 activation after DNA damage.

## Conclusions

Negative feedback in combination with time delay can induce oscillations in cellular networks [25–27]. However, oscillations might be inappropriate in biological systems with adaptive behaviour [34].

Here, we systematically study how design features in combination with time delay tune the response patterns of biochemical networks. To this end, we create a range of models containing an explicit time delay and a DNF differing in several aspects: presence of a nested negative (auto-inhibitory) feedback, presence of mass conservation for a system component and mechanism of DNF, i.e., input-inhibition or output-activation (Fig. 1). The obtained models (Models 1-4) are mathematically described by two-dimensional delay differential Eqs. 1–(4) and subjected to computational and theoretical stability analyses with respect to time delay. The general idea to specifically address the interaction of time delay and design features was that all these design features act on the system stability and response pattern by modifying the time delay threshold, i.e., the bifurcation point, beyond which the system’s stability properties change.

We show that

- nested auto-inhibitory feedbacks and overall delayed negative feedbacks have opposing roles with respect to the characteristic response pattern. Indeed, nested auto-inhibitory feedbacks have the potential to suppress oscillatory behaviour, whereas the increasing strength of the DNF promotes oscillations. Moreover, in oscillatory systems auto-inhibitory feedbacks de-couple amplitude and period of oscillations.
- mass conservation has a stabilizing effect on the system’s equilibrium.
- depending on the parameter set, the type of DNF can also influence the response pattern. We found that input-inhibition can be more stabilizing compared to output-activation.

Thus, biochemical networks have a range of design possibilities shaping both their dynamic as well as their equilibrium properties. Our systematic analysis of different design features allows predicting what kind of biochemical network underlies a certain characteristic response. For example, in oscillatory systems with a long time delay, it is reasonable to assume a limited number of post-translational modifications (mass conservation), no nested feedbacks and a strong overall negative feedback. Whereas adaptive systems with long time delay are likely to harbour nested negative feedbacks and post-translational modifications. Systems with low number of components and short time delay that are meant to oscillate, will need an abrupt negative feedback with low activation threshold, whereas short time delay and a weak negative feedback are good designs principles for adaptive systems.

Our framework of delayed and non-delayed feedbacks can serve to support a design process for novel synthetic gene-regulatory networks. Indeed, our study allows to approximate the value of time delay and the structure of the DNF system for obtaining a certain type of the system dynamics. As an example, we considered p53 system in mammalian cells that contains DNF and is able to produce both oscillatory and adaptive responses depending on the source and strength of DNA damage [37, 41]. Although many studies are dedicated to studying DNA damage response in cells, the purpose of oscillations in p53 system remains unclear [29, 37, 39, 41]. The earlier study [37] suggested that oscillatory behaviour can be advantageous for the p53 system to achieve a trade-off between irreversible biological outcome, e.g., irreversible cell cycle arrest or apoptosis, and insufficiently low levels of p53. Thus, oscillations have been viewed as repetitive repair efforts allowing the system to check after every p53 pulse whether the damage has been properly repaired. Our analysis showed that time delay increases the duration of “Off” states and decreases the duration of “On” states. Additionally, time delay may increase the amplitude and period of oscillations. According to our analysis the auto-inhibitory feedback is able to decouple the amplitude and period of oscillations with respect to time delay. Thus, our study suggests that auto-inhibition and time delay may control oscillations in p53 system. The experimental validation of these predictions may help to better understand the role of p53 oscillations and indicate more efficient treatment of diseases caused by violation of p53 regulation.

## Additional file

Additional file 1 Supporting material. The file contains detailed stability analysis of Models 1-4; theoretical analysis showing how auto-inhibition increases *τ*_*m*_; demonstration how *τ*_*m*_ can be increased by auto-inhibition for the p53 model; details of robustness analysis of the optimal solution for the p53 model; modelling the switch from oscillatory to adaptive response of the p53 system; calculating the duration of “On” and “Off” states of p53 pulses; figure demonstrating the dependence between parameter values of Models 1-4 and *τ*_*m*_; figure with results of Monte-Carlo analysis of Models 1-4 applied to the alternative parameter set; table with the best-fit parameters for the p53 model. (PDF 1044 kb)

## Abbreviations

DNF: delayed negative feedback
ODE: ordinary differential equation
SSR: sum of squared residuals
