# Supplementary material for: How time delay and network design shape response patterns in biochemical negative feedback systems
Source: BMC Syst Biol. 2016 Aug 24;10(1):82. doi: 10.1186/s12918-016-0325-9 (PMC4995745; doi:10.1186/s12918-016-0325-9)
Supplement: Additional file 1 — Supporting material. The file contains detailed stability analysis of Models 1-4; theoretical analysis showing how auto-inhibition increases τ m; demonstration how τ m can be increased by auto-inhibition for the p53 model; details of robustness analysis of the optimal solution for the p53 model; modelling the switch from oscillatory to adaptive response of the p53 system; calculating the duration of “On” and “Off” states of p53 pulses; figure demonstrating the dependence between parameter values of Models 1-4 and τ m; figure with results of Monte-Carlo analysis of Models 1-4 applied to the alternative parameter set; table with the best-fit parameters for the p53 model. (PDF 1044 kb) [file 12918_2016_325_MOESM1_ESM.pdf]

How time delay and network design shape response patterns in biochemical negative feedback systems

Supporting material

Anastasiya Börsch[1,2] and Jörg Schaber[1,*]

[1]Institute for Experimental Internal Medicine, Medical Faculty, Otto-von-Guericke University, Pfälzer Platz 2, Magdeburg 39106, Germany
[2]Biozentrum, University of Basel and Swiss Institute of Bioinformatics, Klingelbergstrasse 50–70, Basel 4056, Switzerland

* j.schaber@web.de

Stability analysis of Models 1-4

In this section, we present the stability analysis of Models 1-4, which is similar to the analysis of DNF systems described in our previous study [1]. The majority of presented formulas can be applied to all models and we do explicitly distinguish them, unless necessary.

We linearised Models 1-4 about their respective equilibrium (C_s, R_s):

$$\begin{pmatrix} \dfrac{dC}{dt} \\ \dfrac{dR}{dt} \end{pmatrix} = \begin{pmatrix} -x & -y \\ 0 & -\beta \end{pmatrix} \begin{pmatrix} C \\ R \end{pmatrix} + \begin{pmatrix} 0 & 0 \\ 1 & 0 \end{pmatrix} \begin{pmatrix} C(t-\tau) \\ R(t-\tau) \end{pmatrix}, \tag{1}$$

where elements x, y are represented in equations (7)-(10) of the main manuscript for Models 1-4, respectively.

Further, we formulated a characteristic transcendental equation of the system (1):

$$P(\lambda, \tau) = (\lambda + x)(\lambda + \beta) + y\, e^{-\lambda\tau} = 0, \text{ where } x, y > 0. \tag{2}$$

According to the lemma from [2], as τ varies, the sum of the multiplicities of zeros of (2) in the open right half-plane $M(\tau)$ can change only if a zero appears on or crosses the imaginary axis. Thus, the only way that $M(\tau) \neq M(\tau')$ for $\tau < \tau'$ is, if there exists a marginal value τ_m between τ and τ', such that $P(\lambda_m, \tau_m) = 0$ and $Re(\lambda_m) = 0$ [3].

Consequently, we presented a root of (2) in the form $\lambda_m = i\,\omega_m$, where $\omega_m > 0$:

$$P(i\,\omega_m, \tau_m) = x\,\beta - \omega_m^2 + i\,(x + \beta)\,\omega_m + y\, e^{-i\,\omega_m \tau_m} = 0.$$

Applying Euler's formula $e^{-i\,\omega_m\tau_m} = \cos(\omega_m\tau_m) - i\sin(\omega_m\tau_m)$ we obtain:

$$x\,\beta - \omega_m^2 + i\,(x + \beta)\,\omega_m + y\,(\cos(\omega_m\tau_m) - i\sin(\omega_m\tau_m)) = 0. \tag{3}$$

Satisfying (3) real and imaginary parts should both be equal to zero:

$$x\,\beta - \omega_m^2 + y\cos(\omega_m\tau_m) = 0, \tag{4}$$

$$(x+\beta)\,\omega_m - y\sin(\omega_m\tau_m) = 0, \tag{5}$$

which is equivalent to:

$$\left(x\,\beta - \omega_m^2\right)^2 = \left(-y\cos(\omega_m\tau_m)\right)^2,$$

$$(x+\beta)^2\,{\omega_m}^2 = \left(y\sin(\omega_m\tau_m)\right)^2.$$

We summed up above equations and applied Pythagorean trigonometric identity:

$$\omega_m^4 + (x^2+\beta^2)\omega_m^2 + x^2\,\beta^2 - y^2 = 0. \tag{6}$$

Equation (6) has real non-zero roots if and only if $x^2\,\beta^2 - y^2 < 0$, which is equivalent to $x\,\beta < y$. Otherwise, τ_m does not exist and the equilibrium (C_s, R_s) of Models 1-4 is absolutely stable. We assumed that $x\,\beta < y$ holds and defined the discriminant D of (6) by

$$D = (x^2+\beta^2)^2 - 4(x^2\,\beta^2 - y^2). \tag{7}$$

Then, we defined roots of (6):

$$\omega_{m\pm}^2 = \frac{1}{2}\left(-x^2 - \beta^2 \pm \sqrt{D}\right).$$

Using the fact that ω_m^2 should be positive, we get:

$$\omega_m^2 = \frac{1}{2}\left(-x^2 - \beta^2 + \sqrt{D}\right),\ \omega_m = \sqrt{\frac{1}{2}\left(-x^2 - \beta^2 + \sqrt{D}\right)}.$$

Further, we substituted the obtained expression for ω_m into (4) and (5):

$$\cos(\omega_m\tau_m) = \frac{1}{2\,y}\left(-(x+\beta)^2 + \sqrt{D}\right), \tag{8}$$

$$\sin(\omega_m\tau_m) = \frac{x+\beta}{y}\,\omega_m > 0. \tag{9}$$

Using (8) and (9) we expressed the value of τ_m in the following form:

$$\tau_m(n) = \frac{1}{\omega_m}\left[\arccos\left(\frac{1}{2\,y}\left(-(x+\beta)^2 + \sqrt{D}\right)\right) + 2\pi n\right],\ n = 0,\ 1,\ 2, ...$$

Since $\sin(\omega_m\tau_m) > 0$ holds, we concluded that if $\left(-(x+\beta)^2 + \sqrt{D}\right) > 0$, then $\arccos\left(\frac{1}{2\,y}\left(-(x+\beta)^2 + \sqrt{D}\right)\right) \in \left(0, \frac{\pi}{2}\right)$, otherwise $\arccos\left(\frac{1}{2\,y}(-(x+\beta)^2 + \sqrt{D})\right) \in (\frac{\pi}{2}, \pi)$. In both cases, the smallest time delay, which causes a purely imaginary pair of roots $\lambda_{1,2} = \pm i\omega_m$, is

$$\tau_m = \frac{1}{\omega_m}\arccos\left(\frac{1}{2\,y}\left(-(x+\beta)^2 + \sqrt{D}\right)\right). \tag{10}$$

Further, we proved the stability of the equilibrium (C_s, R_s) for any $\tau \in [0, \tau_m)$. Indeed, roots of $P(\lambda, 0)$ are either real negative or have negative real part (see above).

Then, according to Lemma from [2], $M(\tau) = 0$ for any $\tau \in [0, \tau_m)$. Hence, for any $\tau \in [0, \tau_m)$ the equilibrium (C_s, R_s) is asymptotically stable.

Now, we prove that a Hopf bifurcation occurs at $\tau = \tau_m$. For this we differentiated the characteristic polynomial (2) with respect to τ and equated it to zero:

$$2\lambda \frac{d\lambda}{d\tau} + (x+\beta)\frac{d\lambda}{d\tau} = y\, e^{-\lambda\tau}\left(\tau \frac{d\lambda}{d\tau} + \lambda\right).$$

Then, we obtained the expression for $\frac{d\lambda}{d\tau}$:

$$\frac{d\lambda}{d\tau} = \frac{y\,\lambda}{e^{\lambda\tau}\,(x+\beta+2\lambda) - y\,\tau}.$$

We substituted $\lambda = i\omega_m$ into the expression for $\frac{d\lambda}{d\tau}$ and applied Euler's formula:

$$\frac{d\lambda}{d\tau}(\tau) = \frac{i\,\omega_m\, y\cos(\omega_m\tau) + \omega_m\, y\sin(\omega_m\tau)}{x+\beta - y\,\tau\cos(\omega_m\tau) + i\,(2\omega_m + \tau y\sin(\omega_m\tau))}.$$

We substituted the expression for τ_m (10), used (8) and (9), multiplied the top and the bottom by the conjugate of the denominator, took real part and obtained:

$$\frac{dRe(\lambda)}{d\tau}(\tau_m) = \frac{\sqrt{D}\,\omega_m^2}{(x+\beta - y\,\tau_m\cos(\omega_m\tau_m))^2 + (2\,\omega_m + \tau_m\, y\,\sin(\omega_m\tau_m))^2} > 0. \tag{11}$$

The positivity of (11) guarantees that the hypotheses of the implicit function theorem hold. Hence, we may conclude that for $\tau \approx \tau_m$ the root of the characteristic polynomial (2) $\lambda_m = i\omega_m$ crosses the imaginary axis from left to the right.

According to Proposition 6.5 from [3], for $\tau \geq \tau_m$ the equilibrium (C_s, R_s) is unstable.

Auto-inhibition increases τ_m

We represented τ_m (10) as a function of x, y and β:

$$\tau_m(x,y,\beta) = f(x,y,\beta)\,g(x,y,\beta), \tag{12}$$

where

$$\begin{aligned} f(x,y,\beta) &= \frac{\sqrt{2}}{\sqrt{-x^2-\beta^2+\sqrt{(x^2+\beta^2)^2+4(y^2-x^2\beta^2)}}} > 0, \\ g(x,y,\beta) &= \arccos\frac{-(x+\beta)^2+\sqrt{(x^2+\beta^2)^2+4(y^2-x^2\beta^2)}}{2y} > 0. \end{aligned}$$

Assuming $x\,\beta < y$ and using D from (7), we obtained derivatives of functions f and g with respect to x and y:

$$\begin{aligned}
\frac{\partial f}{\partial x}(x,y,\beta) &= \frac{\sqrt{2}x\left(\sqrt{D}-(x^2-\beta^2)\right)}{\sqrt{D}\left(\sqrt{D}-x^2-\beta^2\right)^{3/2}} > 0,\\
\frac{\partial g}{\partial x}(x,y,\beta) &= \frac{2(x+\beta)\left(\sqrt{D}-x^2+x\beta\right)}{\sqrt{D}\sqrt{4y^2-\left((x+\beta)^2-\sqrt{D}\right)^2}} > 0,\\
\frac{\partial f}{\partial y}(x,y,\beta) &= -\frac{2\sqrt{2}y}{\sqrt{D}\left(\sqrt{D}-x^2-\beta^2\right)^{3/2}} < 0,\\
\frac{\partial g}{\partial y}(x,y,\beta) &= -\frac{(x+\beta)^2\left(-x^2-\beta^2+2x\beta+\sqrt{D}\right)}{\sqrt{D}y\sqrt{4y^2-\left((x+\beta)^2-\sqrt{D}\right)^2}} < 0.
\end{aligned} \tag{13}$$

Thus, we conclude that $f(x,y,\beta)$ and $g(x,y,\beta)$ are both positive functions, which increase with x and decrease with y. Consequently, $\tau_m(x,y,\beta)$ follows the same pattern. Using the relation between equilibria of models with and without auto-inhibitory feedback and properties of feedback functions F, S_1, S_2 described in the main manuscript, we calculated lower and upper bounds of x and y. Further, we showed how these bounds can be increased or decreased leading to changing in the value of τ_m.

Firstly, we defined the relation between equilibrium components R_s and C_s for Models 1-4. For this we equated the right hand side of the differential equation $\dfrac{dR}{dt}$ to 0:

$$R_s = \frac{1}{\beta}C_s. \tag{14}$$

Further, for each Model 1-4 we equated the right hand side of the differential equation $\dfrac{dC}{dt}$ to 0 and substituted the expression for R_s (14):

$$\underbrace{I\,S_1\left(\frac{1}{\beta}C_s\right)}_{\theta_1}\cdot F(C_s) = \underbrace{\alpha\,C_s}_{\theta_2} \text{ for Model 1,} \tag{15}$$

$$\underbrace{I\,S_1\left(\frac{1}{\beta}C_s\right)(1-C_s)}_{\theta_1}\cdot F(C_s) = \underbrace{\alpha\,C_s}_{\theta_2} \text{ for Model 2,} \tag{16}$$

$$\underbrace{I}_{\theta_1}\cdot F(C_s) = \underbrace{\alpha\,C_s + \delta\,C_s\,S_2\left(\frac{1}{\beta}C_s\right)}_{\theta_2} \text{ for Model 3,} \tag{17}$$

$$\underbrace{I\,(1-C_s)}_{\theta_1}\cdot F(C_s) = \underbrace{\alpha\, C_s + \delta\, C_s\, S_2\left(\frac{1}{\beta}C_s\right)}_{\theta_2} \text{ for Model 4.} \tag{18}$$

Thus, for each model we got the equation defining the equilibrium component C_s in the form:

$$\theta_1 \cdot F(C_s) = \theta_2. \tag{19}$$

According to (19), we find the equilibrium component C_s as the intersection of functions $\theta_1 \circ F$ and θ_2 (see Fig. S1). Since θ_1 is either constant or decreasing function with respect to C and θ_2 is increasing function with respect to C, the equilibrium component C_s always exists and is unique. In case the auto-inhibitory feedback is not present in the system ($F(C) \equiv 1$), the equilibrium component $\widehat{C}_s$ of the model without auto-inhibition is always greater than the equilibrium component C_s of the model with auto-inhibition (see Fig. S1). According to (19), the same conclusion holds for equilibrium components R_s and $\widehat{R}_s$ of models with and without auto-inhibition, respectively.

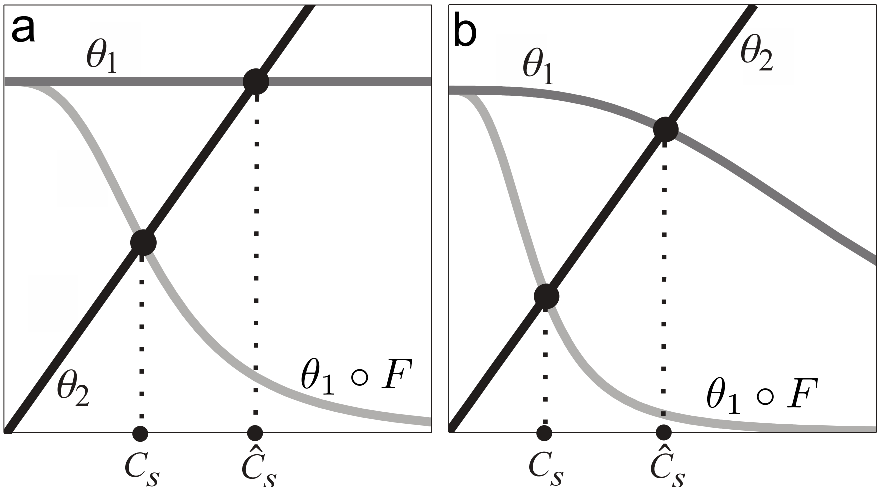

Figure S1 Schematic intersection of functions θ_1, $\theta_1 \circ F$ and θ_2. Functions θ_1 and θ_2 were introduced to find equilibrium components of Models 1-4. **a** How to find equilibrium components C_s, $\widehat{C}_s$ for Model 3. **b** How to find equilibrium components C_s, $\widehat{C}_s$ for Models 1, 2, 4.

Taking into account obtained relations $C_s \leq \widehat{C}_s$ and $R_s \leq \widehat{R}_s$ and properties of feedback functions F, S_1, S_2 described in the main manuscript we obtained following estimations of x and y:

$$\begin{gathered} 0 < \varepsilon_{lb}(|F'(C_s)|) < x < \varepsilon_{ub}(|F'(C_s)|), \\ 0 \leq \sigma_{lb} < y < \sigma_{ub}. \end{gathered} \tag{20}$$

Refer to Table S2 for values of ε_{lb}, ε_{ub}, σ_{lb}, σ_{ub} for each Model 1-4.

Both the lower and upper bound of x, i.e., ε_{lb} and ε_{ub}, are increasing with $|F'(C_s)|$ for Models 1-4. Therefore, we can always increase a given x by choosing an appropriate value for $|F'(C_s)|$. The lower and upper bound of y, i.e., σ_{lb} and σ_{ub}, have non-negative constant values. Consequently, according to (13), we can always increase τ_m by increasing $|F'(C_s)|$.

Taken together, we show how auto-inhibitory feedback allows to modify the range of the interval $[0, \tau_m)$.

Numerical evidence of increasing τ_m by auto-inhibition

The theoretical analysis presented in the section above shows that increasing the slope of the auto-inhibitory function at the equilibrium $|F'(C_s)|$ leads to increasing the marginal time delay τ_m. Here, we suggest an algorithm how to increase $|F'(C_s)|$ and, consequently, τ_m for the p53 model.

As auto-inhibitory feedback function we used a reverse Hill function $F_H(C)$ (Fig. S2a):

$$F_H(C) = \frac{1}{1 + (\kappa\, C)^{\nu}},\ \nu > 1,\ \kappa \geq 0. \tag{21}$$

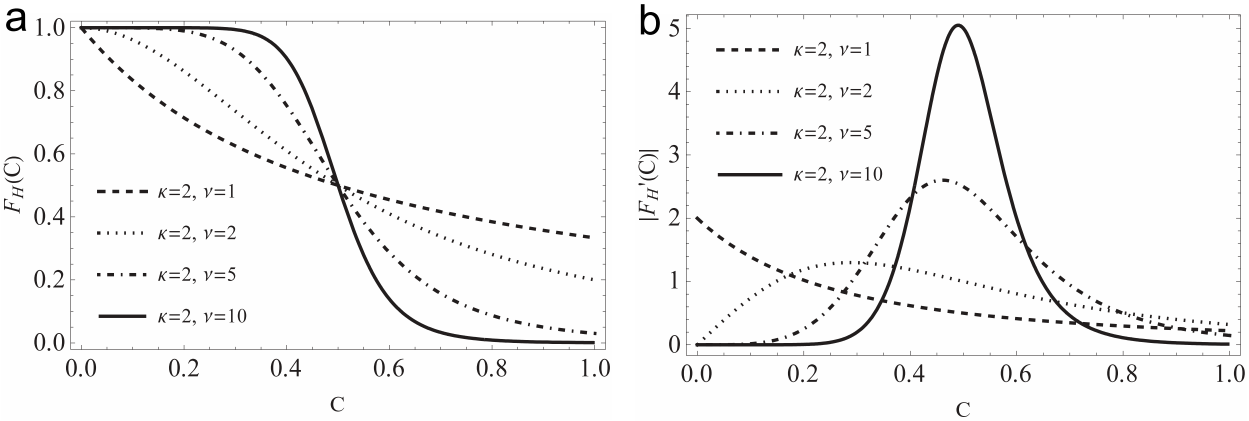

Figure S2 Reverse Hill function. **a** Plot of the reverse Hill function $F_H(C)$ (21). **b** Absolute value of the derivative of the reverse Hill function with the fixed κ and different values of ν.

For the reverse Hill-function, an obvious choice to increase the slope is increasing the Hill-coefficient ν. Therefore, we made ν a free positive parameter, which value we choose. For simplicity we considered only integer values of ν, however the algorithm can be extended to real values of ν. Then we adjusted values of κ and equilibrium (C_s, R_s) and maximized $|F'_H(C_s)|$, which is equivalent to solving $F''_H(C_s) = 0$. Thus, for the fixed value of ν we solved the following system of equations with respect to (C_s, R_s, κ) using fitted parameter values $(\alpha, \beta, \delta, K_m, n)$ from Table S1:

$$\begin{aligned} F''_H(C_s) &= \frac{\nu(\kappa\, C_s)^{\nu}\left((\nu+1)(\kappa\, C_s)^{\nu} - \nu + 1\right)}{C_s^2\left((\kappa\, C_s)^{\nu} + 1\right)^3} = 0, \\ 0 &= I\, F_H(C_s) - \alpha\, C_s - \delta\, C_s\, S_2(R_s), \\ 0 &= C_s - \beta\, R_s. \end{aligned} \tag{22}$$

Then, we quantified τ_m using (12). If it is necessary, we increase ν, solve the system (22) with respect to (C_s, R_s, κ) and quantify τ_m again.

Fig. S3 demonstrates results of application of the algorithm to the p53 model. In Fig. S3a we illustrated the graph of the slope $|F'_H(C)|$ for several values of ν and adjusted values of κ. One can see that $|F'_H(C_s)|$ is increasing, when we increase ν, whereas the value of C_s is only slightly decreasing (indicated by black dots in Fig. S3a). Consequently, τ_m is also increasing with respect to the Hill coefficient ν (Fig. S3b). For $\nu \geq 3$ the equilibrium is asymptotically stable. For $\nu > 8$

the equilibrium is absolutely stable. Thus, the relation between ν, $|F'_H(C)|$ and τ_m demonstrated in Fig. S3 corresponds well to the theoretical analysis made above.

In Fig. S4 we depicted simulations of the p53 model with fitted parameters from Table S1 and synthetically activated auto-inhibitory feedback $F_H(C)$ (21) with $\nu = 3$, $\kappa = 1.73$. One can see that the model with auto-inhibitory feedback produces damped instead of sustained oscillations.

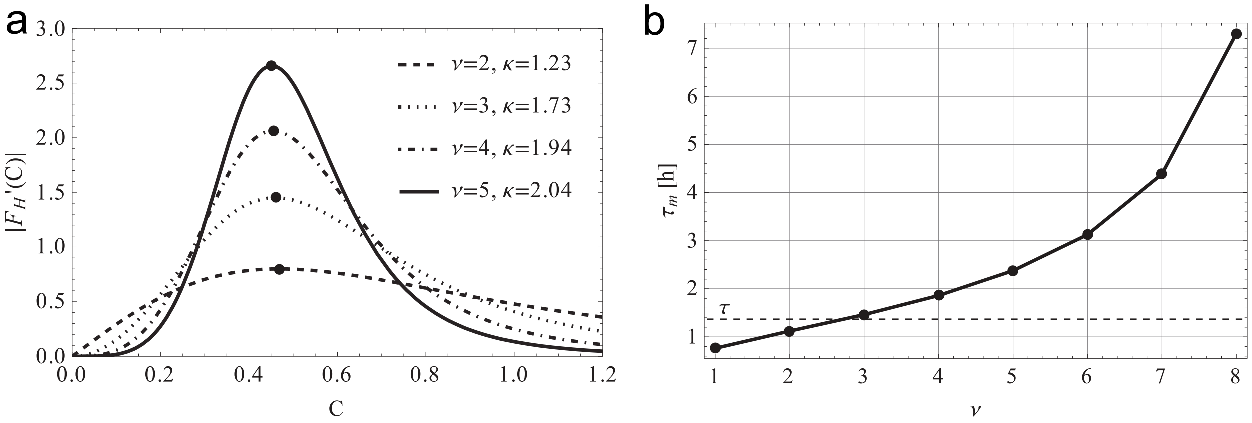

Figure S3 Influence of the synthetically activated auto-inhibitory feedback $F_H(C)$ on the stability of the p53 model. **a** Absolute value of the derivative of $F_H(C)$ for $\nu = 2, 3, 4, 5$ and adjusted values of κ. Dots – absolute values of the derivative at the equilibrium point C_s. **b** Dependency between τ_m and Hill coefficient ν. The dashed line designates the value of the fitted time delay τ.

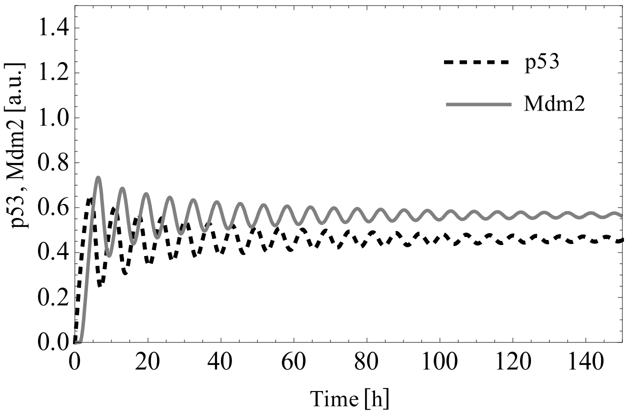

Figure S4 Simulation of the p53 model with synthetically activated auto-inhibitory feedback. Simulation of the p53 model with fitted parameters from Table S1 and synthetically activated auto-inhibitory feedback $F_H(C)$ (21) with $\nu = 3$, $\kappa = 1.73$.

Further, we investigated the stability of the p53 model with and without synthetically activated feedback with respect to several parameters. In Fig. S5a-c we show that increasing the Hill coefficient ν and adjusting κ leads to a decreasing parameter region, where oscillations occur. Fig. S5d demonstrates that τ_m increases with respect to κ for both $\nu = 2$ (dark gray) and $\nu = 3$ (light grey). Thus, these results correspond well to our previous research [4], where we proved for the model with delayed negative feedback that nested negative feedbacks lead to increasing resistance of the

system. In addition, in Fig. S5a we illustrate that τ_m increases with respect to α that indicates the stabilizing property of α and may explain such a small fitted parameter value. On the other hand, τ_m decreases with respect to I (Fig. S5c).

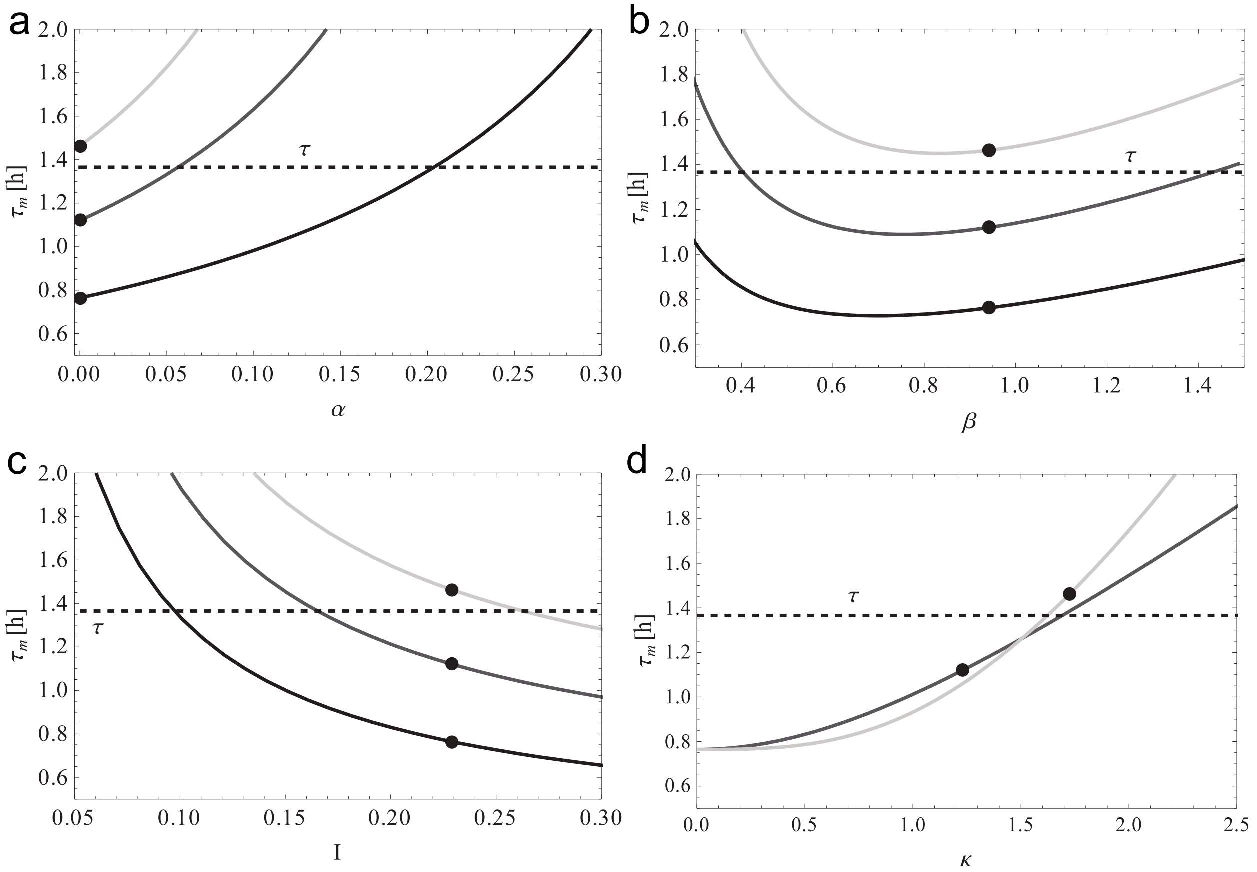

Figure S5 Dependencies between parameters of the p53 model. The p53 model is considered without (black) and with synthetically activated ($\nu = 2,\ \kappa = 1.23$ dark grey, $\nu = 3,\ \kappa = 1.73$ light grey) auto-inhibitory feedback. **a** τ_m and α. **b** τ_m and β. **c** τ_m and I. **d** τ_m and κ. Designations: dots – the fitted value of the parameter used in the p53 model, dashed line – the fitted value of τ (Table S1).

Robustness of optimal solution of p53 model

We analysed the robustness of the optimal solutions for the p53 model with respect to noise. To this end, we randomly sampled parameter values within $\pm 10\%$ of their respective fitted values using a uniform distribution for 100 times. Then we simulated p53 model with perturbed parameters and calculated 0.05 and 0.95 quantiles of obtained simulations (*grey regions* in Fig. S6). Fig. S6 shows that the model solution with fitted parameters is located between 0.05 and 0.95 quantiles for all considered time points.

Further, for each perturbed parameter set we calculated the relative variation of the integral of

the first transient response after the stimulation:

$$var_i = \frac{\left|\int_0^{t_{Int}} C(t)dt - \int_0^{t_{Int}} C_p^i(t)dt\right|}{\int_0^{t_{Int}} C(t)dt} \cdot 100\%,$$

where $C(t)$ corresponds to the model solution with fitted parameters, $C_p^i(t)$ corresponds to the model solution with i^{th} perturbed parameter set ($i = 1, 2, 3, ..., 100$), t_{Int} corresponds to the time of the first minimum after initial stimulation (Fig. S6).

This way, the robustness of both initial activation amplitude and timing of the first transient response, two characteristic measures of system dynamics, can be estimated concomitantly.

As the measure of robustness we calculated the mean value $< var >$ and standard deviation sd of obtained relative variations var_i:

$$< var > \pm sd = 8.7 \pm 6.4\%.$$

One may see that for the considered model values of $< var >$ and sd do not exceed 9%. Thus, the fitted solution turned out to be very robust with respect to noise in the parameters, also indicating that the fitted solution is in a well-defined local minimum.

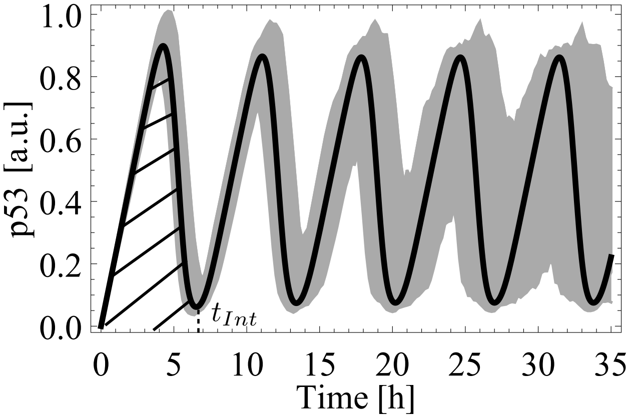

Figure S6 Robustness of the optimal solution of the p53 model. Fitted parameter values of the p53 model were perturbed 100 times and used for model simulations. Gray region: 0.05-0.95 quantiles of simulations of the parametrized model with perturbed parameters. Black solid line: simulations of the p53 model with fitted parameters from Table S1. Dashed areas correspond to integral values $\int_0^{t_{Int}} C(t)dt$ used for calculating relative variations var_i.

Modelling the switch from oscillatory to adaptive response of the p53 system

For modelling the transition from oscillatory to adaptive response of the p53 system we extended the p53 model (3) by including an additional component ATM. The resulting wiring scheme is depicted in Fig. S7.

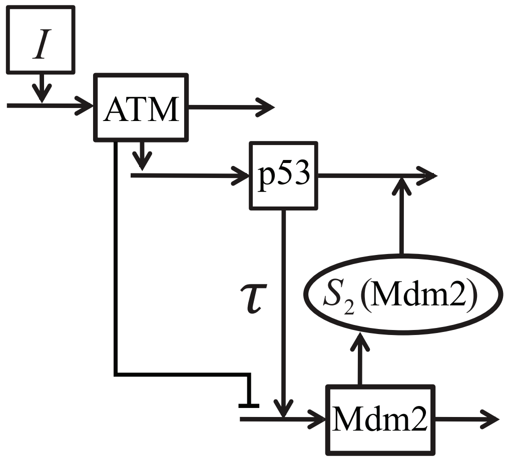

Figure S7 Wiring scheme of the extended p53 model.

We translated the wiring scheme from Fig. S7 into the following system of DDEs:

$$\begin{aligned} \frac{dATM}{dt} &= I - \gamma\, ATM, \\ \frac{dp53}{dt} &= ATM - \alpha\, p53 - \delta\, p53\, \frac{Mdm2^n}{Mdm2^n + {K_m}^n}, \\ \frac{dMdm2}{dt} &= \frac{p53(t-\tau)}{1 + ATM^{n_2}} - \beta\, R. \end{aligned} \tag{23}$$

The extended p53 model (23) was further simulated with parameters from Table S1, $\gamma = 0.05$ and $n_2 = 2$ (see Fig. S8a) and with parameters from Table S1, $I = 1$, $\gamma = 0.05$ and $n_2 = 2$ (see Fig. S8b). As a result, the extended p53 model demonstrates sustained oscillations for low DNA damage ($I = 0.23$) and monotone adaptive response for high DNA damage ($I = 1$).

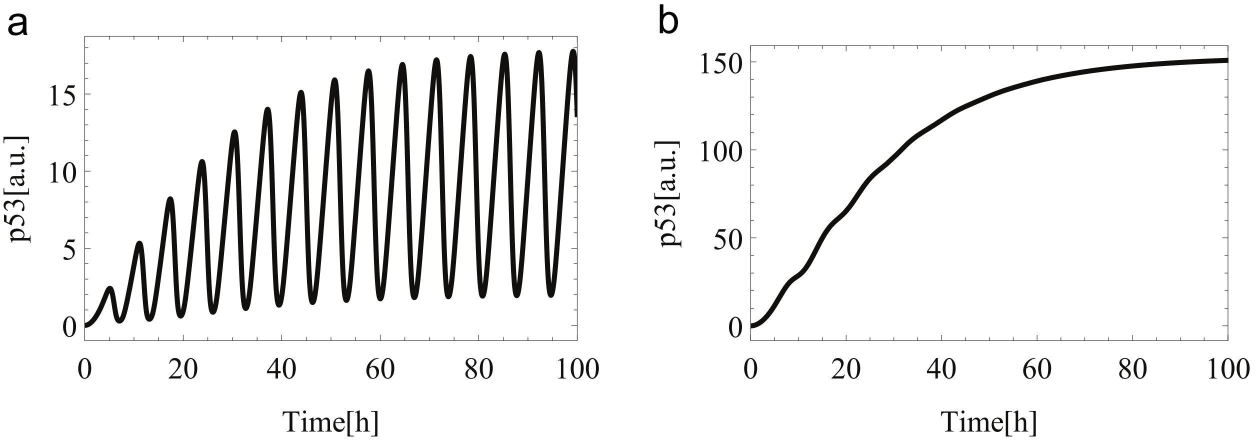

Figure S8 Simulation of the extended p53 model (23). **a** Simulation with parameters from Table S1, $\gamma = 0.05$ and $n_2 = 2$. **b** Simulation with parameters from Table S1, I=1, $\gamma = 0.05$ and $n_2 = 2$.

Calculating the duration of "On" and "Off" states of p53 pulses

For calculating the duration of "On" and "Off" states of p53 pulses we simulated p53 model (3) with parameters from Table S1 for later time points ($t > 400$ h), when the system reached its steady state (see Fig. S9). Then we quantified the mean value between maxima and minima of the simulation curve (see black dashed line in Fig. S9). As a result the simulation curve was split on upper and lower parts with respect to the mean value. We designated the upper part as "On" state and the lower part as "Off" state.

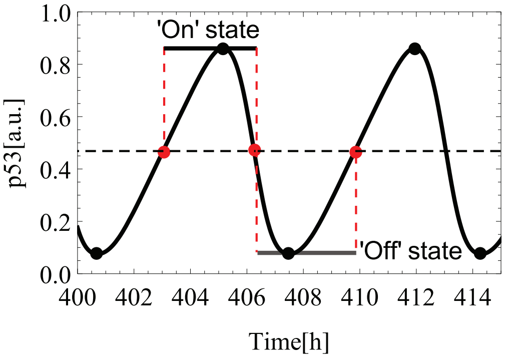

Figure S9 Defining "On" and "Off" states for the p53 response. Black dashed line designates the mean value between maxima and minima of the simulation curve.

Then we checked how different model parameters control the duration of "On" and "Off" states (see Fig. S10). For this we varied parameter values I, τ, K_m, n, α, δ, β, κ and ν one at a time in the range, where p53 model (3) produces sustained oscillations. Then for each varied parameter value we calculated the duration of "On" and "Off" states of the p53 response as described above.

Table S1 Best-fit parameters used for the p53 model from the main manuscript.

Parameters	p53 model
I	0.23 $[a.u.]$
τ	1.37 h
α	$5.9 \times 10^{-13}\,\mathrm{h}^{-1}$
β	$0.94\,\mathrm{h}^{-1}$
δ	$176\,\mathrm{h}^{-1}$
K_m	$2\,\mu$M
n	5
$C(0)$	0 $[a.u.]$
$R(0)$	0 $[a.u.]$
SSR	2.16

SSR - the sum of squared residuals

Table S2 Lower and upper bounds of x and y for Models 1-4.

Model 1
$\varepsilon_{lb} = I\, S_1(\widehat{R}_s)|F'(C_s)| + \alpha,$<br>$\varepsilon_{ub} = I\, S_1(0)|F'(C_s)| + \alpha,$<br>$\sigma_{lb} = 0,$<br>$\sigma_{ub} = I\ \max_{R\in[0,\widehat{R}_s]} |S_1'(R)|.$
Model 2
$\varepsilon_{lb} = I\, S_1(\widehat{R}_s)\,(1 - \widehat{C}_s)|F'(C_s)| + \alpha,$<br>$\varepsilon_{ub} = I\, S_1(0)\,[1 + |F'(C_s)|] + \alpha,$<br>$\sigma_{lb} = 0,$<br>$\sigma_{ub} = I\ \max_{R\in[0,\widehat{R}_s]} |S_1'(R)|.$
Model 3
$\varepsilon_{lb} = I\,|F'(C_s)| + \alpha + \delta\, S_2(0),$<br>$\varepsilon_{ub} = I\,|F'(C_s)| + \alpha + \delta\, S_2(\widehat{R}_s),$<br>$\sigma_{lb} = 0,$<br>$\sigma_{ub} = \delta\, \widehat{C}_s\ \max_{R\in[0,\widehat{R}_s]} |S_2'(R)|.$
Model 4
$\varepsilon_{lb} = I\,(1 - \widehat{C}_s)|F'(C_s)| + \alpha + \delta\, S_2(0),$<br>$\varepsilon_{ub} = I\ [1 + |F'(C_s)|] + \alpha + \delta\, S_2(\widehat{R}_s),$<br>$\sigma_{lb} = 0,$<br>$\sigma_{ub} = \delta\, \widehat{C}_s\ \max_{R\in[0,\widehat{R}_s]} |S_2'(R)|.$

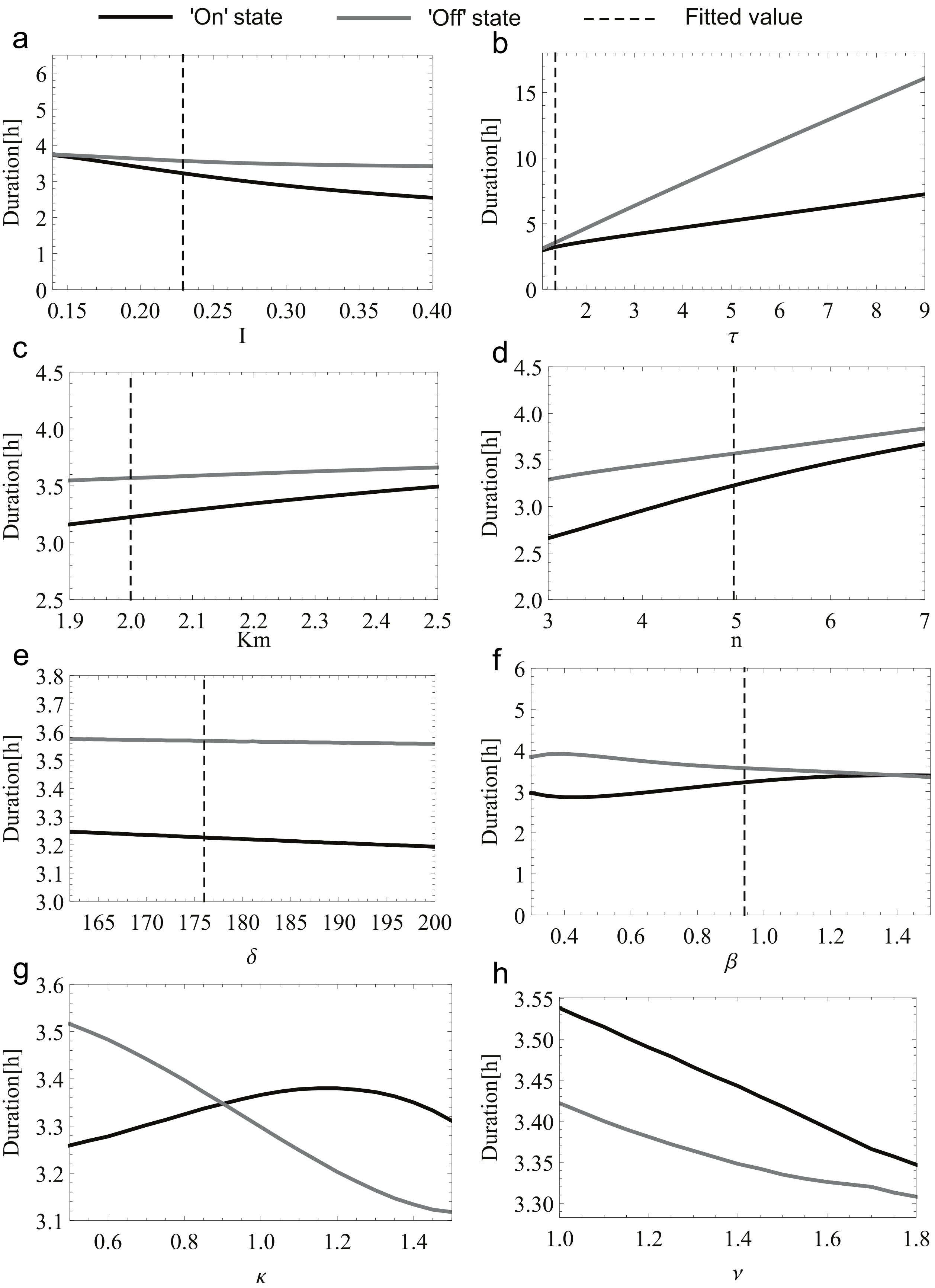

Figure S10 Dependency between parameter values of the p53 model (3) and duration of "On" and "Off" states. **a** I, $\kappa = 0$. **b** τ, $\kappa = 0$. **c** K_m, $\kappa = 0$. **d** n, $\kappa = 0$. **e** δ, $\kappa = 0$. **f** β, $\kappa = 0$. **g** κ, $\nu = 3$. **h** ν, $\kappa = 1.73$.

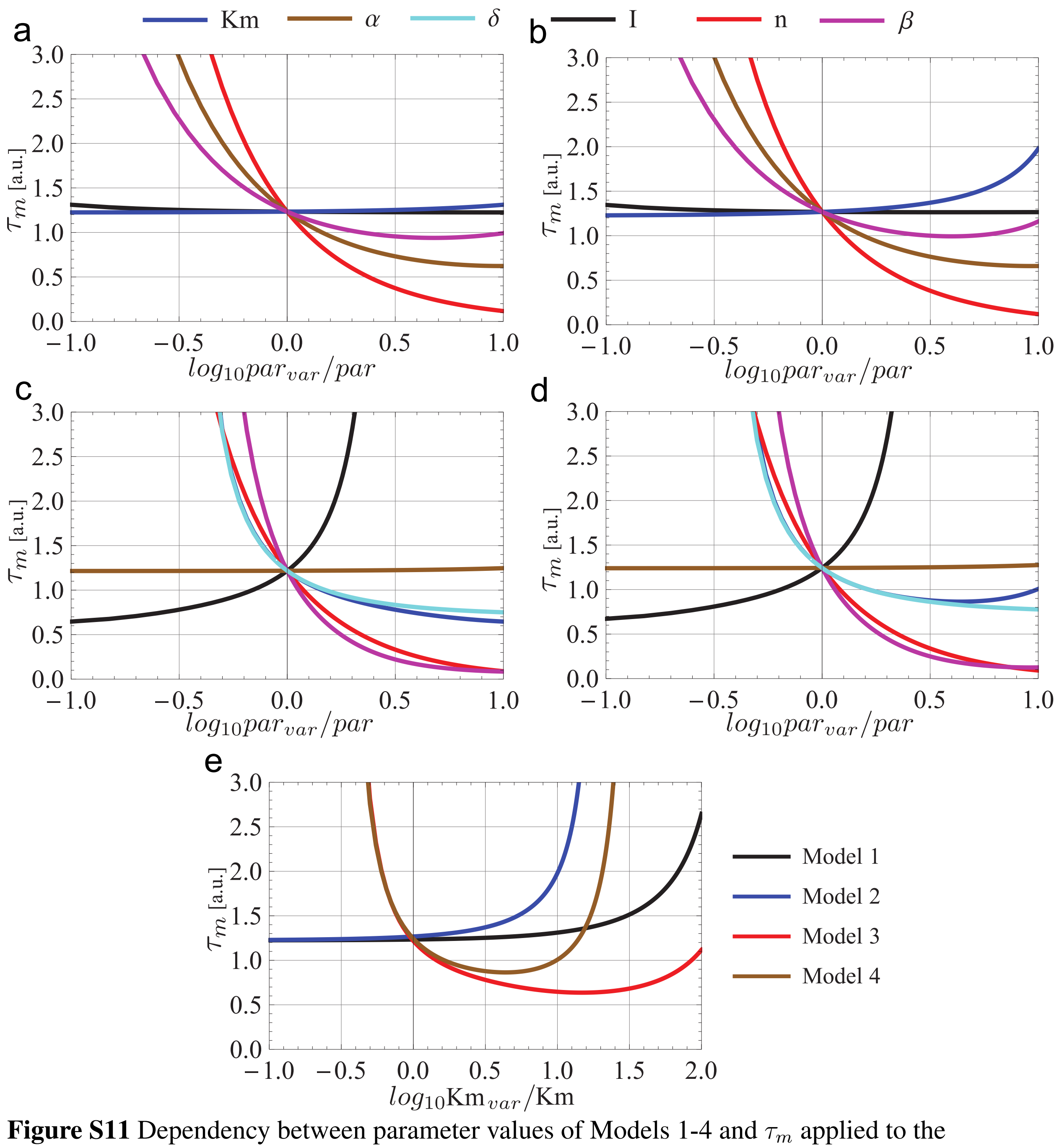

Figure S11 Dependency between parameter values of Models 1-4 and τ_m applied to the parameter set $I = 0.87$, $\alpha = 0.11$, $\beta = 0.17$, $\delta = 58.2$, $n = 12.77$, $K_m = 0.23$. **a** For Model 1. **b** For Model 2. **c** For Model 3. **d** For Model 4. **e** Dependency between the parameter K_m and τ_m for Models 1-4. With par_{var} and par we designated varied and initial chosen parameter values, respectively. For Figs. **a-d** we varied the value of each parameter in the range from 0.1 to 10 times of its respective chosen value leaving the rest parameter values fixed. For Fig. **e** we varied the parameter K_m in the range from 0.1 to 100 times of its respective chosen value.

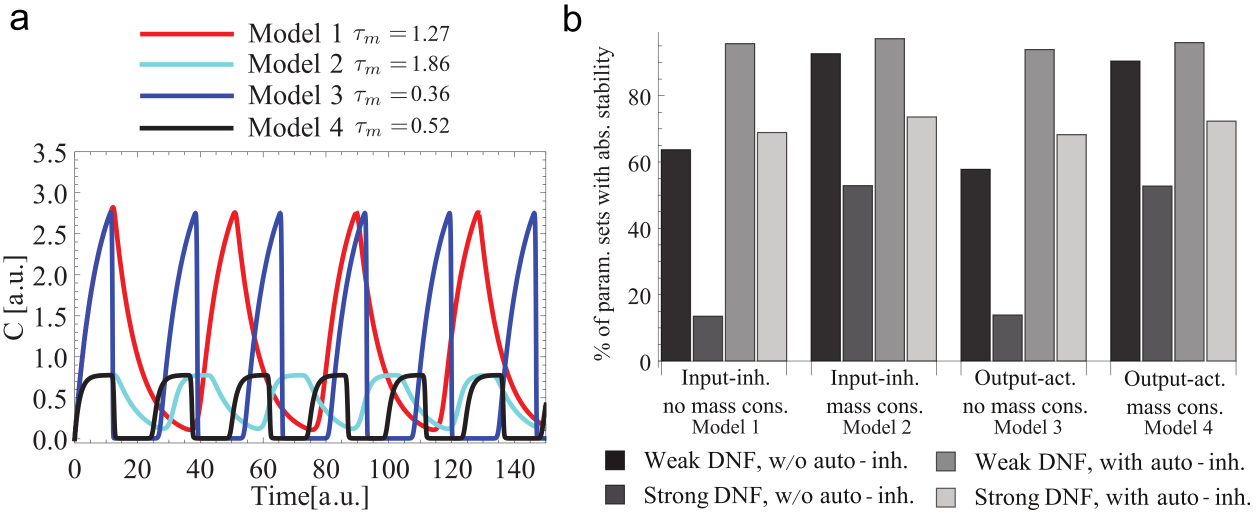

Figure S12 Results of Monte-Carlo analysis of Models 1-4 applied to the second parameter set $I = 0.48$, $\alpha = 0.14$, $\beta = 0.44$, $\delta = 83.71$, $n = 10$, $K_m = 0.9$, $\tau = 10$ without auto-inhibition ($\kappa = 0$). **a** Simulation of Models 1-4. **b** Stability analysis of Monte-Carlo simulations of Models 1-4. Model parameters were randomly sampled 10000 times in the certain range. The range was defined according to assumptions about model characteristics: strength of DNF (strong or weak) and presence of auto-inhibition. The percentage of parameter sets, which induced absolute stability, was quantified.

References

1. Lapytsko, A., and J. Schaber, 2016. The role of time delay in adaptive cellular negative feedback systems. *Journal of Theoretical Biology* 398:64–73. http://dx.doi.org/10.1016/j.jtbi.2016.03.008.

2. Cooke, K. L., and Z. Grossman, 1982. Discrete delay, distributed delay and stability switches. *Journal of Mathematical Analysis and Applications* 86:592 – 627.

3. Smith, H., 2010. An Introduction to Delay Differential Equations with Applications to the Life Sciences. Texts in Applied Mathematics. Springer Science+Business Media, LLC.

4. Schaber, J., A. Lapytsko, and D. Flockerzi, 2014. Nested autoinhibitory feedbacks alter the resistance of homeostatic adaptive biochemical networks. *J. R. Soc. Interface* 11:20130971.
